# Atypical Case of Combined Types I and II Scleredema Mimicking Morphea on Histopathology

**DOI:** 10.7759/cureus.34077

**Published:** 2023-01-23

**Authors:** Sarah A Alhunaif, Abeer Alsarheed, Raghad Almutairi, Ghaida Almutairi

**Affiliations:** 1 Dermatology, King Abdulaziz Medical City - National Guard Health Affairs, Riyadh, SAU

**Keywords:** atypical presentation, scleredema of buschke, type 2 scleredema, type 1 scleredema, scleredema

## Abstract

Scleredema is a rare sclerotic skin disorder that typically develops in conjunction with diabetes, monoclonal gammopathy, or infection and commonly involves the neck, back, trunk, and arms. Scleredema can be categorized into three types according to its cause. The local examination of the lesion is characterized by non-pitting induration that follows a symmetrical spread with sparing of the hands and feet. We present a case of a 19-year-old female patient that presented to the outpatient clinic complaining of facial skin lesions that have been progressive for four years. The skin lesions were found to involve the neck, back, trunk and both arms sparing the hands and there was no systemic involvement of the disease. The patient is non-diabetic but reported frequent attacks of tonsillitis over the past months. Initially, punch biopsy showed no signs of scleredema; however, repeated biopsy at follow-up confirmed the presence of mucin deposits which are suggestive features of scleredema. Due to the similarities with various other diseases, the diagnosis requires clinical and histopathological exclusion which makes the diagnosis quite challenging. It almost always starts in the neck but can present initially in rare cases from the face spreading symmetrically. Close follow-up and continuous monitoring are necessary for systemic organ involvement.

## Introduction

Scleredema is an uncommon skin disease with an unidentified pathophysiology characterized by symmetrical, non-pitting stiffening and induration of the skin, which is caused by an excessive increase in mucin deposition between the thickened and widened collagen bundles commonly involving the upper back, shoulder, and neck. Based on its associations with post-infection often streptococcal, monoclonal gammopathy, and diabetes mellitus, scleredema is traditionally categorized into three types [1]. Scleredema was first described by Curizo in 1752 and was later elaborated on and well-defined by Buschke in 1902 [2]. Various risk factors such as infection, drugs, genetic disposition, and inflammatory processes may be responsible for accounting for scleredema [2]. Physical examinations can present with annular rash and transient erythema with complaints of difficulty in mastication due to the involvement of the tempomandibular joint. In addition, the skin shows wood-like poorly defined lesions and is associated with non-pitting indurations [3]. Scleredema is diagnosed through clinical signs and symptoms, and a definitive diagnosis is made through histopathology. This usually shows a normal or slightly thinner epidermis and fewer elastic fibers and thick, swollen collagen bundles, especially type 1 collagen, in the deep reticular dermis, which is separated by mucopolysaccharide deposits. Mucin deposits are made up mostly of hyaluronic acid, which is not sulfated. The number and shape of fibroblasts are usually normal. The subcutaneous fat is replaced by thick collagen fibers, and the skin adnexa is pushed up [4].

Scleredema was classified into three types according to Graff in 1968. The first type (type I) is the most common subgroup affecting 55% of all cases of scleredema and is almost observed only in the pediatric and young adult age groups and is characterized by a febrile streptococcal throat infection preceding the abrupt \skin condition by two to three weeks. Meanwhile, type II scleredema is a slowly progressive condition and is associated with hematological abnormalities such as monoclonal gammopathy of undetermined significance, multiple myeloma, and chronic myeloid leukemia. Type III involves diabetic patients and is therefore referred to as scleredema diabeticorum. It occurs in around 20% of all cases of scleredema and is caused by long-lasting poorly controlled insulin-dependent diabetes mellitus. Furthermore, types II and III usually have extracutaneous involvement with them affecting the tongue, heart, esophagus, spleen, liver, and lungs [2].

Morphea on the other hand is a localized form of scleroderma and affects primarily just the skin. Lesions are usually small and mostly only one lesion is observed. However, it can also spread in a variety of morphologies, including guttate, nodular, subcutaneous, and linear. Morphea is a rare condition that affects nearly three times as many women as it does men. Morphea is normally asymptomatic, with the exception of the occasional itch and, in rare cases, pain. It is characterized by linear atrophy and/or hardness of the skin, subcutis, and muscle and bone involvement. Lesions start as an erythematous or violaceous linear indurated moderate atrophic plaque and then proceed to hypopigmented or depigmented sclerotic deep furrows [5-7]. The signs of morphea that are seen in histopathology include the line sign, the cookie-cutter sign, and the square biopsy sign, additionally, a high number of eccrine glands and the presence of interstitial mucin are also mentioned in various studies [8].

Treatment of scleredema is supportive in targeting the causative underlying condition as there is no specific treatment for treating the skin condition itself [9]. Nonetheless, phototherapy is usually the first line of treatment used by many clinicians and if not an effective trial of intravenous immunoglobulin is recommended [2] We present a case of scleredema that showed different histopathological characteristics than that of typical scleredema mimicking morphea.

## Case presentation

**Figure 1:**
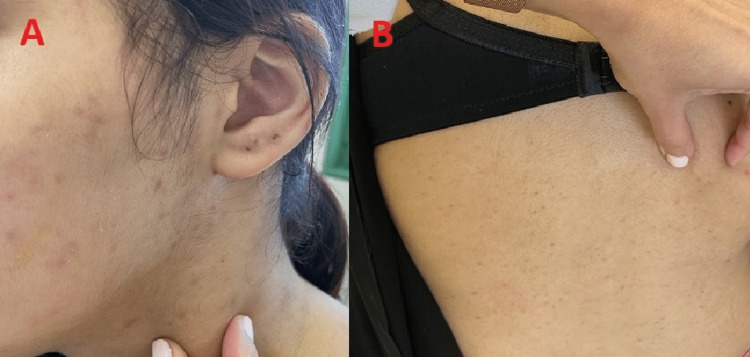
(A) Diffuse sclerosis (dark pigmentation) on the neck. (B) Diffuse sclerosis on the back (tiny spots of dark pigmentation).

**Figure 2:**
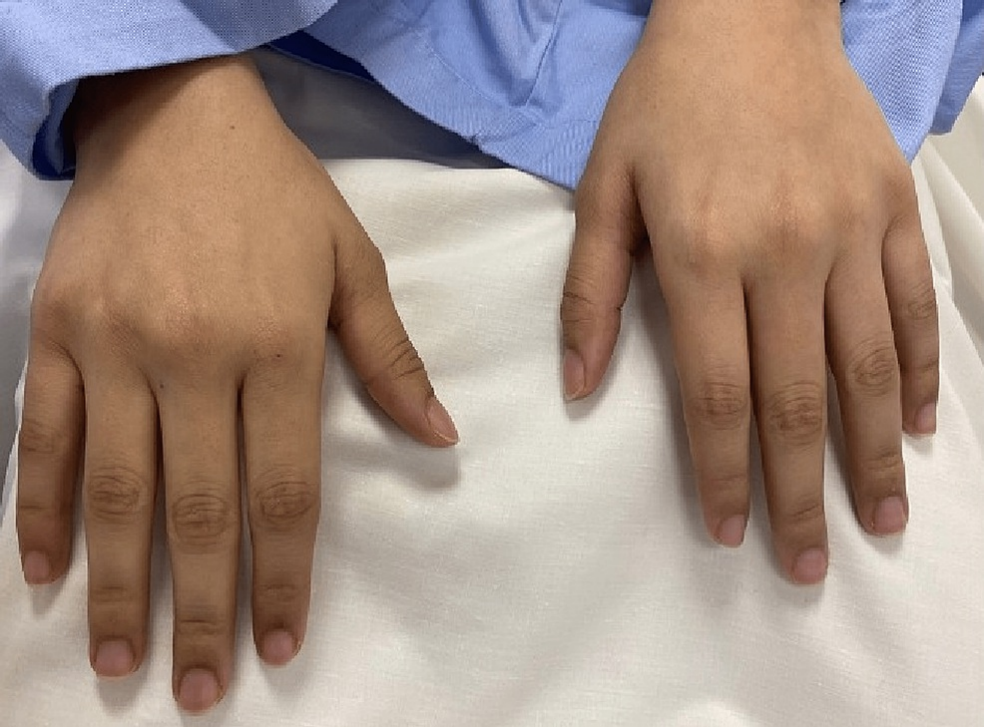
Hands of the patient are spared from the lesions.

A 19-year-old medically free female presented to the primary health care clinic complaining of skin tightness over the face and forearms for the past four years which was first noticed by her family three to four years ago. There was no history of cough, shortness of breath, skin rash, itching, photosensitivity, ulcers, joint pain, fever, weight loss, or fingertips changes. Clinically, the patient was afebrile and vitally stable with diffuse sclerosis on both forearms and face. There were no signs of redness, inflammation or discharge. Upon questioning, the patient stated that the skin sclerosis started on the face and progressed distally to both forearms with no exacerbating or alleviating factors. The patient was transferred to the rheumatology clinic for further evaluation on suspicion of connective tissue disease (CTD). Laboratory Investigations showed a normal complete blood count while C-reactive protein (CRP), rheumatoid factor and Antinuclear Antibody (ANA) were negative. Furthermore, chest x-ray and echocardiography were normal with no signs of any CTD or any other abnormality. As a result, the case was transferred to the dermatology clinic where the patient did not follow up immediately. Three months later, the patient returned to the dermatology department as a transferred case with the same presentation. On physical examination of the skin lesions, inspection showed there was bilateral forearm induration extending from the neck to the back and anterior trunk with dark discoloration (Figures 1A, 1B). On palpation, the skin lesions were thick with no other abnormality. There were no signs of microstomia, telangiectasia, Raynaud’s phenomenon, acral pigmentation, digital ulcers, peau d'orange appearance or pitted scars while the hands and feet were spared with normal nail capilloscopy (Figure 2).

**Figure 3:**
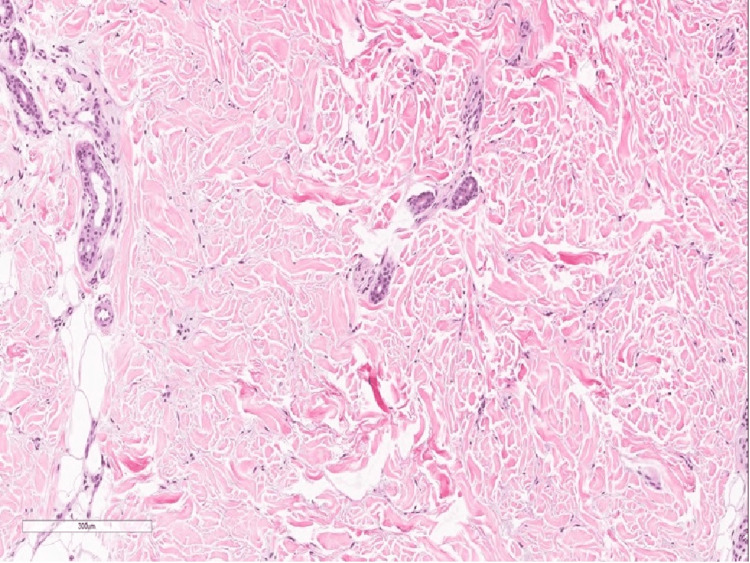
Histopathology slide of a 4-mm punch biopsy from the right forearm showing mildly spaced collagen bundles (magnification x40).

**Figure 4:**
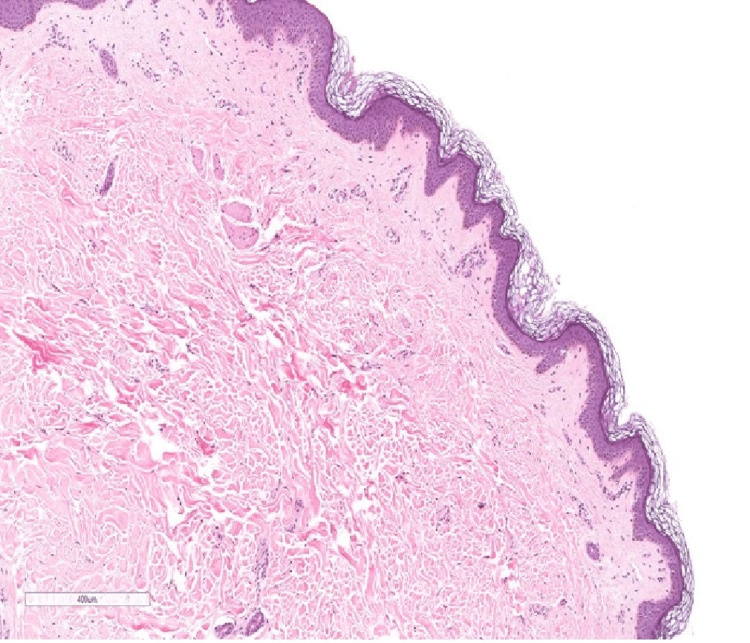
Second biopsy shows mild mucin deposition between the collagen (magnification x40).

Full investigation panel was ordered and showed normal complete blood count, thyroid function, kidney function and liver function tests. Regarding inflammatory profile, Erythrocyte Sedimentation Rate (ESR) was slightly elevated (50 mm/hr) while her CRP was 13 mg/L. Furthermore, ANA, anti-double stranded DNA (anti-dsDNA), rheumatoid factor and cyclic citrullinated peptide were negative while her SCL 70 was 25 U/mL (< 25 IU = Negative; 25-30 IU = Borderline Positive; 30-60 IU = Low Positive; 60-200 IU = Positive; > 200 IU = Strong Positive). Chest x-ray was performed and magnetic resonance imaging (MRI) was sought to discover the extent of the lesion. The MRI reported an increased signal intensity in flexural muscles otherwise the myofascial plane was unremarkable. Initially, exclusion of scleredema was based on the fact that there was sparing of the hands and feet, normal nail capilloscopy, no acral involvement, no Raynaud’s phenomenon and no telangiectasia. Meanwhile, based on the MRI finding eosinophilic fasciitis was excluded with supporting evidence that there was no peau d'orange on pinching and that the lesion was not severe. A 4-mm punch biopsy from the right forearm was performed and revealed a mildly spaced collagen bundles without any sign of mucin deposition (Figure 3). Neither loss of adnexal structures nor thickening of collagen bundles was observed. Therefore, the patient was prescribed methotrexate 10 mg once per week as well as folic acid 1 mg once per day, which was started initially by her rheumatology visit. Regarding her follow up, the patient was still having the same complaint. A decision to take a second punch biopsy was sought in order to confirm the diagnosis of morphea and exclude other causes. The biopsy showed deposition of mucin and increased mast cells suggestive of scleredema (Figure 4).

Furthermore, the patient reported recurrent attacks of tonsillitis. On examination of her throat, we found enlarged tonsil with no signs of infection. Furthermore, an Antistreptolysin O (ASO) titer was found to be elevated and was referred for possible tonsillectomy as the cause of scleredema. To determine the type of scleredema, hemoglobin A1c (HbA1c) and paraprotein work was ordered and showed normal levels. Therefore, based on the previous infection, the patient was determined to have both type 1 and type 2 scleredema. Treatment options including surveillance post-tonsillectomy, phototherapy, methotrexate, intravenous immunoglobulin was discussed with the patient. She chose to continue with medical treatment and observation with monitoring and continuous follow up but never followed up.

## Discussion

Scleredema is a sclerotic skin disease that can be divided into three types. Type 1 is often preceded by an acute fever or inflammatory disease and is very common in middle-aged women [5]. Type 2 often carries a long progressive course without a history of infection or diabetes [5]. Meanwhile, type 3 is often associated with diabetes and obesity and is common among middle-aged men [5]. In comparison, our patient had a prolonged slow course of the disease for four years but also had repeated attacks of throat infection making this presentation suggestive of both type 1 and 2. Even though scleredema is often a diagnosis of exclusiveness, it is often presented as non-pitting induration of the skin with overlying erythema. It starts at the neck and spreads in a symmetrical fashion to the back, trunk, and forearms with sparing of the hands and feet [1]. Similarly, our patient presented with skin lesions affecting the neck, back, and arms with no involvement of the hands and feet. However, the lesions also started from the face which is atypical in cases of scleredema. Moreover, in typical cases of scleredema, the skin often appears as peau d'orange, which was not present in our patient. In the literature, other unusual atypical presentations reported the presence of bilateral eyelid edema and periorbital edematous swelling [6,7]. In comparison, such features were not present in our patient making the provisional diagnosis of eosinophilic fasciitis or morphea.

A histopathological examination of the lesion is not needed to confirm the diagnosis. However, most cases of scleredema show thickening of the collagen bundles separated by clear mucin-filled space and the presence of mucin deposits in most cases [3]. In comparison, our patient’s first punch biopsy was not suggestive of scleredema which showed mildly spaced collagen bundles with no signs of mucin deposits. However, the second biopsy which was taken due to the persistence of the disease showed features suggestive of scleredema with mucin deposits observed accordingly. Moreover, no laboratory investigation or imaging can confirm cases of scleredema making it a diagnosis based on exclusion by clinical history and histopathology. In addition, a history of repeated attacks of pharyngitis and tonsillitis is often associated with type 2 scleredema. According to Cron and Swetter, 58% of cases of type 2 scleredema are preceded by the acute febrile illness of ß-hemolytic streptococcus origin [8]. However, other infectious causes have been reported in children associated with type 2 scleredema such as influenza, mumps, and measles [8]. Our case showed no bacterial growth in her throat swab culture despite reporting frequently repeated attacks of tonsillitis in the past.

There is no definitive treatment for cases of scleredema. According to the European dermatology forum S1-guideline, a modified Rodnan scale, which is a measurement of skin thickness, should be utilized to determine the severity of the skin involvement to keep the progress of the treatment compliance and response [4]. Moreover, treatment can be different in each type of scleredema. In cases of type 1, antimicrobial agents can be effective in eliminating the infectious organism with a good prognosis [4]. Furthermore, phototherapy is often used as a first-line treatment modality by clinicians due to its relatively low-cost and reported effectiveness [2]. Other therapies include cyclosporine and steroids while in severe cases intravenous immunoglobulin can be used [1].

Type 1 scleredema has the best prognosis of all types due to its infectious root which can be eliminated using antimicrobial agents. In type 2 scleredema, the prognosis is poor, and careful follow-up and observation are warranted as systemic involvement may occur. It has also been reported to be related to other malignancies such as multiple myelomas in about 1% of cases [9]. Type 3 also has a poor prognosis similar to type 2 requiring continuous monitoring and follow-up of the diabetic status and metabolic state of the patient [4]. According to Rongioletti et al., sleep apnea is common in cases of type 3 scleredema and should be thoroughly investigated [2]. Our patient’s unique presentation has some features suggestive of both type 1 and type 2. However, she is not diabetic and did not report any of the common findings found in the three subgroups of the disease. The course of the disease originated in the face which is atypical in cases of scleredema. There was no bacterial throat growth despite having repeated and frequent attacks of tonsillitis which is suggestive of type 1 scleredema. Four years of chronicity of the lesions makes it more likely to be type 2. In the end, we believe this case is an atypical case combining both type 1 and type 2 of scleredema.

## Conclusions

Scleredema is a rare sclerotic skin disease that is associated with skin lesions. Unusual presentation of the disease can be presented as a prolonged history of skin sclerosis involving the face, neck, back, trunk and extremities with a history of repeated tonsillitis or pharyngitis. Clinical histopathology should be repeated for confirmation and exclusion of other scleredemal diseases. Tonsillectomy can be suggested along with medical treatment and continuous monitoring and follow-up.
